# Ectopic, hepatic GLP-1R agonism enhances the weight loss efficacy of GLP-1 analogues

**DOI:** 10.1016/j.molmet.2026.102327

**Published:** 2026-02-05

**Authors:** Jonathan D. Douros, Megan Capozzi, Aaron Novikoff, Jacek Mokrosinski, Barent DuBois, Joseph Stock, Rebecca Rohlfs, Mikayla Anderson, Dominika J. Jedrzejcyk, Svend Poulsen, Erik Oude Blenke, Tomas Dago, Kasper Huus, Peder L. Nørby, Sune Kobberup, Marita Rivir, Joyce Sorrell, Stephanie A. Mowery, Daniel J. Drucker, David A. D'Alessio, Jonathan E. Campbell, Timo D. Müller, Diego Perez-Tilve, Brian Finan, Patrick J. Knerr

**Affiliations:** 1 Novo Nordisk Research Center Indianapolis, Indianapolis, IN, USA; 2 Duke Molecular Physiology Institute, Duke University, Durham, NC, USA; 3 Institute for Diabetes and Obesity, Helmholtz Munich, Neuherberg, Germany; 4 German Center for Diabetes Research (DZD), Neuherberg, Germany; 5 Novo Nordisk Research Center Seattle, Seattle, WA, USA; 6 Novo Nordisk A/S, Måløv, Denmark; 7 Department of Pharmacology and Systems Physiology, University of Cincinnati College of Medicine, Cincinnati, OH, USA; 8 Lunenfeld Tanenbaum Research Institute, Mt. Sinai Hospital, Toronto, ON, Canada; 9 Walther-Straub Institute for Pharmacology and Toxicology, Ludwig-Maximilians-University (LMU) Munich, Germany

**Keywords:** GLP-1, Obesity, Pharmacology, Energy expenditure, Glucagon

## Abstract

**Objectives:**

Unimolecular triagonists drive substantial weight loss in patients with obesity by engaging the glucagon-like peptide 1 receptor (GLP-1R) and glucose dependent insulinotropic polypeptide receptor (GIPR) to reduce food intake (FI) and the hepatic glucagon receptor (GcgR) to enhance energy expenditure (EE). However, their development has been challenged by deleterious cardiovascular (CV) effects, including increased heart rate (HR), elongated QTc, and arrhythmia mediated by GcgR agonism. GLP-1R mono-agonists on the other hand improve both obesity and CV outcomes with negligible effects on EE. We sought to imbue peptide GLP-1R agonists with an EE enhancing effect by combining them with ectopic GLP-1R expression and agonism in hepatocytes.

**Methods:**

We used an adeno-associated virus (AAV) to induce the expression of a functional, liver-specific GLP-1R combined with traditional peptide agonist treatment to drive greater body weight loss via reduced energy intake and increased energy expenditure.

**Results:**

Agonism of the ectopic GLP-1R with either semaglutide, a cAMP biased GLP-1R analogue (NNC5840), or a dual GLP-1R/GIPR agonist in wild-type (WT) diet induced obese (DIO) mice led to enhanced EE and improved weight loss compared to peptide agonist treatment alone.

**Conclusions:**

This represents a novel mechanism for achieving poly-pharmacology to treat obesity.

## Highlights

• A *Glp1r* encoding AAV induces expression of a functional receptor in mouse livers.
• Endogenous GLP-1R does not mediate semaglutide clearance.
• Ectopic GLP-1R mediates semaglutide clearance.
• Ectopic, hepatic *Glp1r* plus semaglutide enhances weight loss in mice.
• Ectopic, hepatic *Glp1r* plus a dual incretin agonist enhances weight loss in mice.

## Graphical abstract

**Figure:**
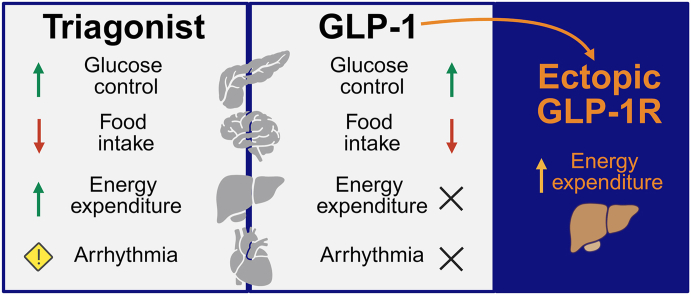

## 1 Introduction

Triple agonists of the GLP-1R, GIPR, and GcgR drive profound weight loss in patients with obesity [[1], [2], [3], [4]]. While the full complement of mechanisms underlying these effects remains to be fully characterized, it is clear that pharmacologic activation of central GLP-1R [5] and GIPR [6,7] populations reduces FI, while hepatic GcgR agonism promotes EE [8,9] in rodents. Unlike their mono- and dual-incretin receptor agonist predecessors, which improve CV function [[10], [11], [12], [13]], development of GcgR activating compounds has been challenged by the increased HR and arrhythmia induced by chronic GcgR agonism [2,3,14,15]. Therefore, we sought to enhance the safe and efficacious weight lowering capacity of GLP-1R and dual GLP-1R/GIPR agonists by augmenting their FI lowering effect with an ability to enhance energy expenditure. To accomplish this, we took advantage of intrinsic similarities and differences in GLP-1R and GcgR. Both receptors couple to Gαs and utilize cAMP as a second messenger. However, only GcgR, not GLP-1R, is expressed in hepatocytes where its pharmacologic activation drives increases in energy expenditure. However, the chronotropic effect of GcgR agonism is linked, at least in rats, to action in the sinoatrial node [16]. Thus, we hypothesized that ectopically expressing and subsequently activating a GLP-1R in hepatocytes would lead to enhanced EE and improved weight loss with no additional increase in HR.

## 2 Methods

### 2.1 Adeno-associated virus (AAV)

The AAV serotype 8 construct was generated by Vector Builder with the *mGlp1r* construct downstream of a liver specific human α-antitrypsin promoter.

### 2.2 Lipid nanoparticle (LNP)

LNP formulations were prepared using a NanoAssemblr Ignite microfluidic mixer (Precision Nanosystems) by mixing at a 3:1 volumetric ratio (aqueous:ethanol) and 12 mL/min total flow rate. Lipid solutions were made in pure ethanol at a molar ratio of [50:10:38.5:1.5] for [ionizable lipid:DSPC:cholesterol:DMG-PEG2000] at a total lipid concentration of 12.5 mM. The ionizable lipid (heptadecan-9-yl 8-((2-hydroxyethyl)(8-(nonyloxy)-8-oxooctyl)amino)octanoate) was synthesized as previously described [17]. DSPC, cholesterol and DMG-PEG2000 were purchased from Sigma–Aldrich. mRNA solution was prepared using nuclease free reagents with final buffer concentration of 25 mM acetate (Sigma–Aldrich) and corresponding to a N/P ratio of 6 when mixed with the lipid excipients.

After microfluidic mixing, LNPs were transferred to 10 kDa MWCO Slide-A-Lyzer Dialysis Cassettes (ThermoFisher Scientific) and dialyzed overnight against 20 mM Tris (Sigma–Aldrich), 8% (w/vol) sucrose (Sigma–Aldrich), pH 7.4. After dialysis, the formulation was sterilized via filtration through a 0.2 μm syringe filter and concentrated by centrifuging at 1000×*g* in Amicon Ultra centrifugal filter unit with a 100 kDa MWCO (Millipore Sigma). After concentration, the formulation was diluted to a final concentration of 0.3 mg/mL mRNA with 20 mM Tris, 8 % (w/vol) sucrose, pH 7.4. LNPs were characterized by dynamic light scattering using a Zetasizer Nano (Malvern Panalytical); the particle diameter (Z-Ave) was 70.7 nm with a PDI of 0.11. mRNA concentration and encapsulation efficiency measurement were performed using Quant-iT RiboGreen reagent (Invitrogen); the encapsulation efficiency was 95.3 % in the final formulation.

### 2.3 mRNA design

*Mus musculus* glucagon-like peptide 1 receptor (*mGlp1r*) (NM_021332.2) coding sequence was codon optimized for expression in murine cells. mRNA was designed to start with GGGA prior to 5′UTR derived from a TOP gene. Two stop codons were added downstream of the *mGlp1r* coding sequence to ensure translation termination. A 70 nt-long poly(A) tail was added to the end of albumin gene 3′UTR.

### 2.4 mRNA synthesis

mRNA was enzymatically, *in vitro* transcribed from double-stranded, PCR-amplified DNA template. Single-stranded DNA template including T7 promoter was chemically synthesized (IDT) and PCR-amplified with SuperFi II PCR Master Mix (ThermoFisher Scientific) with reverse primer containing 70 nt-long dT repeated fragment (IDT). Amplified IVT template was transcribed using a T7 enzyme mix containing T7 polymerase, RNase Inhibitor and inorganic pyrophosphatase (ThermoFisher Scientific). Uridine (U) was substituted with modified uridine analogue, N1-methyl-pseudouridine (N1meψ) (TriLink). The reaction was incubated 4 h at 37 °C with 350 rpm mixing. Following IVT, capping mixture containing Vaccinia Capping Enzyme, Vaccinia mRNA Cap2′-O-Methyltransferase, S-adenosyl-methionine (SAM), and GTP (ThermoFisher Scientific) was added, and reaction was incubated 1 h at 37 °C with 350 rpm mixing. IVT template was removed from reaction by digestion with RNase-free DNase I (ThermoFisher Scientific). mRNA was purified applying Carboxylic Acid Dynabeads for RNA Purification (ThermoFisher Scientific) according to manufacturer's protocol. mRNA quality, integrity and purity were assessed by capillary electrophoresis and A260/280 ratio respectively.

### 2.5 Peptide synthesis

All peptide agonists utilized in these studies were produced according to protocols that have been described in detail elsewhere [8]. Briefly, peptides were constructed using automated Fmoc-base solid-phase peptide synthesis. Following completion of the peptide backbone, acylation with a fatty acid-based protractor was achieved using an orthogonal protecting group strategy on the appropriate lysine sidechain. The peptide was then cleaved from resin, purified by reversed-phase high-performance liquid chromatography, and lyophilized to dryness. Dry peptide was dissolved in an aqueous buffer containing 50 mM phosphate, pH 7.4, 70 mM sodium chloride, and 0.05% Tween 80 for use in animal studies.

### 2.6 In vitro assays

The *in vitro* assays used to characterize GLP-1R internalization induced semaglutide, NNC5840, and the dual GLP-1R/GIPR agonist have been described in detail elsewhere [18]. Briefly, the GLP-1R internalization bioluminescent resonance energy transfer assay was established by measuring loss of baseline resonance energy transfer between an intracellular plasma membrane marker GFP-CAAX and hGLP-1R-RLUC8 or hGIPR-RLUC8 following ligand administration. Transfections were performed using 500 ng of GFP-CAAX DNA and 300 ng of the respective RLUC8-tagged GPCR DNA per well in a 6-well plate. To assess the ability of the *Glp1r* LNP to induce GLP-1R expression *in vitro*, 50 ng LNP was added to 5000 BHK CRE-Luc reporter cells in 24 microtiter plate well replicates. Following 18 h incubation at 37 °C and 5% CO_2_, a 12-point titration of semaglutide was added to the cells in duplicates, resulting in a final volume per well of 25 μL. After 4 h incubation at 37 °C and 5% CO_2_, 12.5 μL detection reagent (Steady-GLO, E2510, Promega) was added to each well and luminescence was detected for each well using a multimode plate reader (EnVision 2105, PerkinElmer).

### 2.7 Animal studies

All animal studies were performed at University of Cincinnati in accordance with approved IACUC protocols. Mice were given *ab libitum* access to water and a 58% fat, high-sugar diet (D12331, Research Diets) for at least 12 weeks to achieve a diet-induced obese state. Animals were housed 3–4 per cage, exposed to a controlled 12 h/12 h light–dark cycle at room temperature (22 °C).

### 2.8 GLP-1R KO mouse studies

For studies with DIO GLP-1R KO mice [19], male animals (mean BW 39.7g) were randomized to treatment groups (n = 3/group) based on starting body-weight. Animals were treated with *mGLP-1R* expressing AAV (10^11^ or 10^12^ GC/mouse) or control AAV (10^12^) at day −7 via tail vein injection. Animals were allowed to recover from day −7 to 0. On day 0 the semaglutide challenge was performed (described below). Body weight and food intake was assessed from day 0 to day 7 with no active treatment. From day 13–33, animals received daily subcutaneous (SC) injections of semaglutide (3 nmol/kg) in all groups. Body weight and food intake were assessed every other day. On day 27, body composition was assessed (described below). Animals were sacrificed and tissues were collected on day 34 for qPCR assessment of GLP-1R mRNA expression (described below). Body weight loss on day 33 was correlated with hepatic GLP-1R mRNA expression.

### 2.9 Proglucagon knockout mice

The proglucagon (ProG) KO mouse line has been validated in previous reports [20,21]. Briefly, ProG KO and wild-type control mice were treated with either control or *mGlp1r* expressing AAV 7 days prior to experiments. We then performed either IPGTT, oral glucose tolerance tests, or mixed nutrient tolerance tests as previously reported [22,23].

### 2.10 Semaglutide challenge

The semaglutide challenge in DIO GLP-1R mice was performed by fasting mice for 6 h prior to the experiment, then administering semaglutide (3 nmol/kg) via SC injection at t = 0. Blood glucose was measured from the tail vein via handheld glucometer over a 6 h time course.

### 2.11 Body composition

Whole body fat and lean mass were measured using nuclear magnetic resonance (EchoMRI, TX).

### 2.12 Weight loss studies in wild type mouse studies

Male C57Bl6 mice were randomized and evenly distributed to test groups (n = 8 per group) according to body weight. Animals received tail vein injection of the control AAV or *mGLP-1R* expressing AAV at a dose of 10^12^ GC per mouse on day −7 for each study. Semaglutide, NNC5840, and dual GLP-1R/GIPR agonist treatment began on day 0 for each study. Treatments were administered via daily SC injection for the duration of the study; dosage is indicated within each figure. Dose escalation regimens for each peptide were determined based on previously published studies [8]. Body weight and food intake was measured every other day throughout the study.

**Figure 1:**
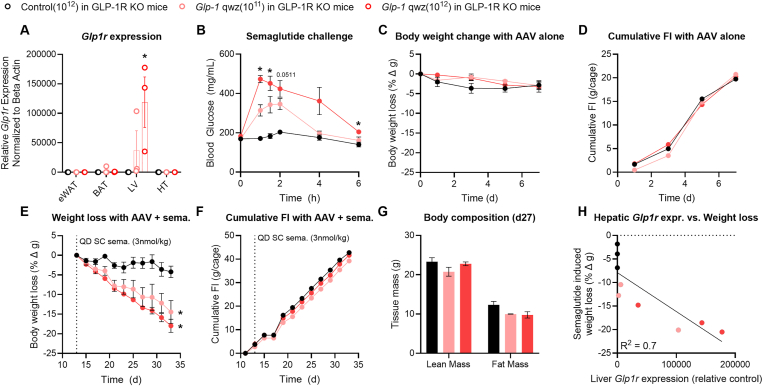
**A *Glp1r* encoding AAV induces expression of a functional receptor in the liver of DIO GLP-1R knockout mice.** Global GLP-1R knockout mice on HFD were dosed I.V. with a control AAV (black bars or circles) or mouse *Glp1r* expressing AAV at 10^11^ genome copies (GC) per mouse (light red bars or circles) or 10^12^ GC per mouse (dark red bars or circles). (**A**) *Glp1r* gene expression relative to beta-actin in the epididymal white adipose tissue (eWAT), brown adipose tissue (BAT), liver (LV), and hypothalamus (HT). (**B**) Blood glucose after a single semaglutide dose (3 nmol/kg). (**C,D**) Body weight change and cumulative food intake (FI) in response to the *Glp1r* expressing AAV alone dosed on day 0. (**E,F,G**) Body weight change, cumulative FI, and body composition (day 27) in response to the *Glp1r* expressing AAV + semaglutide (QD, SC 3 nmol/kg started day 13). (**H**) Correlation between hepatic *Glp1r* expression and semaglutide mediated weight loss. All data are presented as mean ± SEM. ∗ indicates a p-value ≤0.05 compared to control AAV-treated animals or control AAV-treated groups. Notations under/over time points indicate significant difference at that time point determined by 2way ANOVA with Dunnet's test (panel A) or Tukey multiple comparisons test (all others). Notations to the right of the data indicate a significant group effect determined by 2way ANOVA with Tukey multiple comparisons.

**Figure 2:**
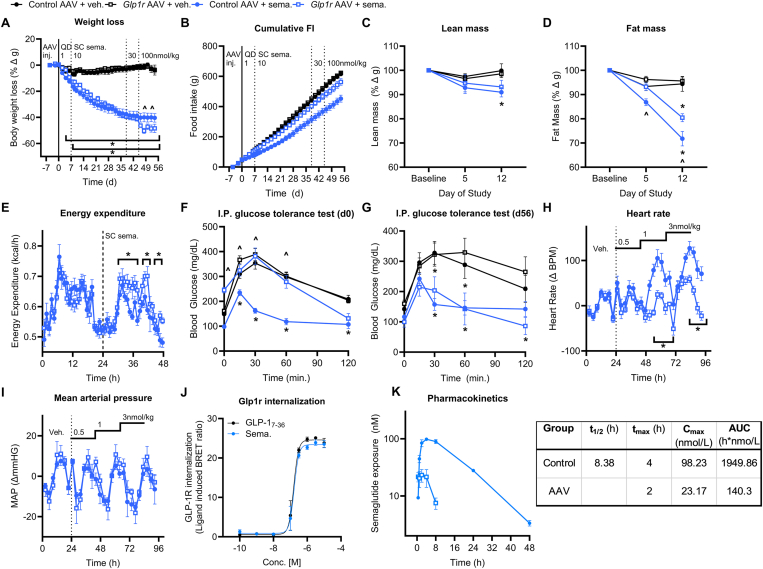
**Coupling ectopic, hepatic *Glp1r* expression and semaglutide treatment enhances weight loss in DIO mice.** WT DIO mice were dosed with a *Glp1r* expressing AAV (10^12^ GC) on day −7, then with semaglutide (dose escalated from 1 to 100 nmol/kg) from day 0–56. (**A,B**) BW loss and cumulative FI. (**C,D**) Lean and fat mass loss on days 0, 5 and 12. (**E**) Acute EE response to semaglutide (3 nmol/kg) in WT DIO mice dosed with either control or *Glp1r* AAV. (**F,G**) Blood glucose during IPGTTs performed on day 0 (F) and day 56 (G). (*H,I*) Heart rate and mean arterial pressure assessed in control AAV and *Glp1r* AAV treated mice in response to semaglutide (0.5, 1, and 3 nmol/kg SC doses). (**J**) *In vitro* GLP-1R internalization induced by native GLP-1 or semaglutide. (**K**) *In vivo* PK profile for semaglutide in WT DIO mice dosed with either control AAV (closed circles) or *Glp1r* expressing AAV (10^12^ GC, open circles). All data are presented as mean ± SEM. ∗ indicates a p-value ≤0.05 compared to respective control animals. ^ˆ^ indicates a p-value ≤0.05 between control AAV + semaglutide and *Glp1r* AAV + semaglutide groups. Notations under/over time points indicate significant difference at that time point determined by 2way ANOVA with Tukey multiple comparisons test. Notations to the right of the data indicate a significant group effect determined by 2-way ANOVA with Tukey multiple comparisons.

**Figure 3:**
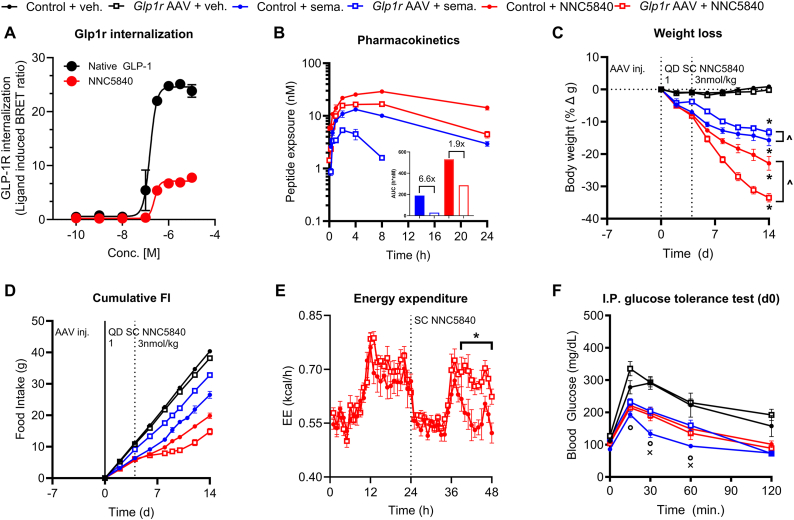
**A GLP-1R agonist that exhibits limited receptor internalization can obviate the PK liability of hepatic *Glp1r* expression.** (**A**) *In vitro* GLP-1R internalization induced by native GLP-1 and NNC5840. (**B**) *In vivo* PK profile for semaglutide or NNC5480 in WT DIO mice dosed with either control or *Glp1r* expressing AAV (10^12^ GC). (**C,D**) Weight loss and cumulative FI during a 14 day treatment regimen with either vehicle, semaglutide, or a NNC5480 in WT DIO mice dosed with either control AAV or *Glp1r* expressing AAV (10^12^ GC). (**E**) Acute EE response to NNC5480 (3 nmol/kg) in WT DIO mice dosed with either control or *Glp1r* AAV. (**F**) Blood glucose during an I.P. glucose tolerance test performed on day 0. All data are presented as mean ± SEM. ∗ indicates a p-value ≤0.05 compared to respective control animals. ^ˆ^ indicates a p-value ≤0.05 between groups as indicated. ^ο^ indicates a p-value ≤0.05 for all groups compared to their respective controls. ^x^ indicates a p-value ≤0.05 between control AAV + semaglutide and *Glp1r* AAV + semaglutide groups. Notations under/over time points indicate significant difference at that time point determined by 2way ANOVA with Tukey multiple comparisons test. Notations to the right of the data indicate a significant group effect determined by 2way ANOVA with Tukey multiple comparisons.

**Figure 4:**
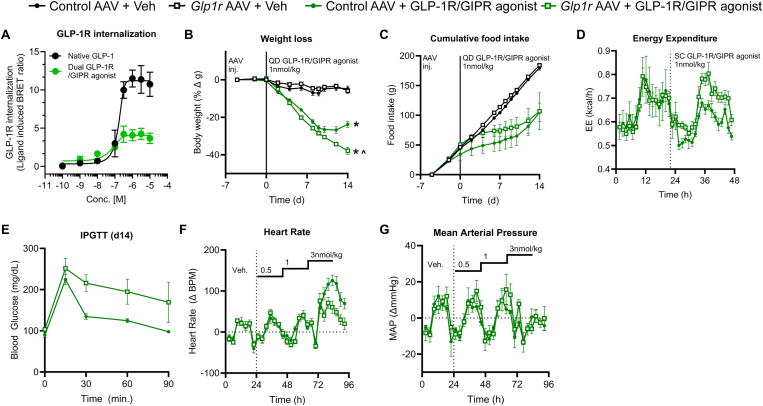
**The weight lowering efficacy of a dual incretin agonist is enhanced in the presence of ectopic, hepatic *Glp1r* expression.** (**A**) *In vitro* GLP-1R internalization for native GLP-1 and a dual GLP-1R/GIPR agonist. (**B,C**) Weight loss and cumulative FI during a 14 day treatment regimen with either vehicle or a dual GLP-1R/GIPR agonist at 1 nmol/kg in control AAV or *Glp1r* AAV treated mice. (**D**) Acute EE response to a dual GLP-1R/GIPR agonist (1 nmol/kg) in WT DIO mice dosed with either control or *Glp1r* AAV. (**E**) Blood glucose during an IPGTT on day 14 of the study. (**F,G**) Acute HR and MAP response to a dual GLP-1R/GIPR agonist in control AAV and *Glp1r* AAV treated mice. All data are presented as mean ± SEM. ∗ indicates a p-value ≤0.05 compared to respective control animals. ^ˆ^ indicates a p-value ≤0.05 between control AAV + GLP-1R/GIPR agonist and *Glp1r* AAV + GLP-1R/GIPR agonist groups. Notations under/over time points indicate significant difference at that time point determined by 2way ANOVA with Tukey multiple comparisons test. Notations to the right of the data indicate a significant group effect determined by 2way ANOVA with Tukey multiple comparisons.

### 2.13 IPGTT

Mice were fasted at the onset of the light phase for 6h prior to the experiment. Treatment compounds were injected at t = −1 h to allow for SC absorption of the compound into circulation prior to injection of a glucose bolus (2 g/kg, intraperitoneal (ip), 20% dextrose in 0.9% NaCl). Blood glucose was measured via handheld glucometer (Abbot Freestyle) over a 2 h time course as outlined.

### 2.14 Indirect calorimetry

Hepatic GLP-1R expression was induced by AAV injection in DIO WT studies as outlined above. Energy expenditure was assessed as previously reported [24]. Briefly, animals were singly housed in the indirect calorimetry systems (TSE Phenomaster/Labmaster, TSE Systems, Chesterfield, MO, USA) for up to 14 days and allowed to acclimate for up to 5 days before measurements began, including QD SC vehicle injections and monitoring baseline body weight and food intake. During the study, mice were either monitored during SC administration of QD compounds or given single injections and measured during the washout period. Body weight and food intake were also monitored.

### 2.15 Cardiovascular radiotelemetry

Male C57Bl6 mice (8–10 weeks old) were anesthetized with isoflurane and surgically implanted with pressure transmitters (PA-C10, DSI), with the catheter inserted into the left carotid artery and the body of the transmitter between the outer skin layer and abdomen wall in the left flank of the mouse. After recovering, they were singly housed and monitored in their home cages. Mice were given control and experimental AAV virus via tail vein injections at least 7 days prior to CV study. Transmitters were turned on via magnets and SC vehicle injections were administered 2 h after the onset of the light phase for baseline measurements. At t = 24 h, mice were grouped if needed and SC compound injections began. Stressors were allowed only from 9:00 AM to 11:00 AM during the light phase, with continuous sampling throughout the day. Measurements of pulse pressure, arterial pressures, and heart rate values were extracted with the DataQuest A.R.T. 4.1 software and averaged into 3-hour bins for analysis.

### 2.16 Gene expression

Gene expression was assessed in the liver, hypothalamus, white adipose tissue, and brown adipose tissue of GLP-1R KO mice given either control or *mGLP-1R* expressing AAV via qPCR protocols as reported elsewhere. The tissue samples were processed in a tissue lyser and RNA was extracted from the samples following the RNeasy MiniKit (Qiagen, Cat. No. 74106) and DNase I treatment (Qiagen, Cat. No. 79256*)*. Using the iScript kit (Bio-Rad, 1708890), cDNA was synthesized. The Taqman probes used are against Glp1r (ThermoFisher, Mm00445292_m1) as the gene of interest, and Beta Actin (ThermoFisher, 4352341E*)* as the endogenous control. Plates were read with the QuantStudio 5 system, and the resulting values were used to calculate the ΔΔCT, normalized to Beta Actin.

### 2.17 Pharmacokinetic studies

For pharmacokinetic studies in wild-type and GLP-1R KO mice, animals were dosed subcutaneously with semaglutide or NNC5840 as described above. Plasma concentrations of NNC5840 and semaglutide were determined by liquid chromatography-tandem mass spectrometry (LC-MS/MS) using a multiple reaction monitoring method. Briefly, plasma proteins were precipitated by mixing plasma samples with 6 volumes of methanol containing internal standard in micronic 1.5 mL microcentrifuge tubes*,* followed by centrifugation for 20 min at 13,000×*g*. The supernatant was transferred to a 96-well plate, diluted with water containing 0.1% formic acid, and mixed thoroughly. Diluted samples were injected into the LC-MS/MS system. The chromatographic separation was performed on a Waters I-class LC system using a Waters Acquity UPLC BEH C18 column (1.0 mm × 50 mm, 1.7 μm) with gradient elution of 0.1% formic acid in water (mobile phase A) and 0.1% formic acid in acetonitrile (mobile phase B) at a flow rate of 0.3 mL/min with a column temperature of 60 °C. The mass spectrometric detection was performed on a Thermo Scientific Quantiva triple quadrupole system with electrospray ionization in positive ion mode. Pharmacokinetic profiles were assessed according to previously reported methods [25]. Briefly, plasma concentration–time profiles were analysed by a non-compartmental method (Pharsight Phoenix WinNonLin v.6.4). The terminal half-life (t_1/2_), maximum plasma concentration (C_max_), time for maximum plasma concentration (T_max_), and AUC from zero to last (AUC_0-t_) were determined. Criteria for estimation of t_1/2_ were at least three concentration–time points in the terminal phase not including Cmax, with an R^2^ ≥ 0.85.

## 3 Results

**Supplemental Figure 1:**
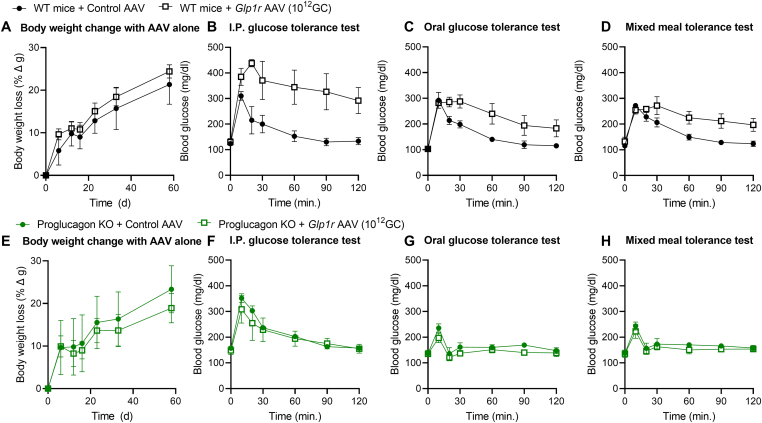
Lean WT mice do not show a baseline body-weight phenotype in response to the ectopic *Glp1r*. Either lean, chow fed WT (A-D) or proglucagon KO (E-H) mice were injected with the *Glp1r* expressing AAV (10^12^ GC) on day 0. (A,E) Body weight change in response to the AAV alone; (B,F) blood glucose during an I.P. glucose tolerance test; (C,G) blood glucose during an oral glucose tolerance test; (D,H) blood glucose during a mixed meal tolerance test. All data are presented as mean ± SEM.

We utilized a *mGlp1r*-encoding AAV8 with a human α-antitrypsin promoter dosed via tail-vein injection to drive liver specific expression (*Glp1r* AAV). In lean WT mice, the *Glp1r* AAV treatment alone did not affect body weight (BW; Supplemental Fig. 1A and E) but did worsen glucose control (Supplemental Figs. 1B,C,D). This trend toward glucose dysregulation is mediated by endogenous proglucagon products activating the ectopic GLP-1R in hepatocytes and recapitulates what is observed with acute glucagon administration. Indeed, this glucose excursion phenotype is lost in proglucagon knock-out mice (Supplemental Figs. 1F,G,H). It should be noted that the α-antitrypsin promotor may drive expression in cells/tissues beside hepatocytes. However, the only phenotype seen in mice treated with the Glp1r expressing AAV alone was a worsened glucose control reminiscent of GCGR agonism. While this outcome in lean mice is deleterious, the model does not represent the pathophysiology we aim to treat. Furthermore, these mice show no food intake, energy expenditure, or body-weight phenotype, suggesting that any off-target expression contributes minimally to the effects presented here. Collectively, these data serve as an indication that the *Glp1r* AAV drives expression of a functional GLP-1R on the liver that mimics GcgR.

We treated DIO GLP-1R KO mice with the *Glp1r* AAV at doses of 10^11^ or 10^12^ genome copies (GC) per mouse (Figure 1A–H) in order to avoid confounding effects of endogenous GLP-1R. Gene expression for *Glp1r* was dose-dependently elevated by *Glp1r* AAV treatment in the liver, but not in the white adipose tissue (eWAT), brown adipose tissue (BAT), and hypothalamus (HT; Figure 1A). There is notable variance in *Glp1r* expression in the AAV treated groups given the sample size (n = 3) and imperfect dose-to-gene expression proportionality across groups. Blood glucose was not acutely reduced in control AAV treated GLP-1R KO mice after a single injection of GLP-1R mono-agonist semaglutide (3 nmol/kg), as expected given the lack of GLP-1R in these animals (Figure 1B). However, semaglutide acutely increased blood glucose in GLP-1R KO mice given the *Glp1r* AAV, consistent with the ectopic, hepatic expression of a functional GLP-1R that mimics GcgR when agonized (Figure 1B). The *Glp1r* AAV groups exhibited no change in BW or food intake (FI) with AAV treatment alone (Figure 1C,D). On initiation of once-daily subcutaneous semaglutide injections (day 13, Figure 1E,F), both *Glp1r* AAV treated groups lost BW, but showed little reduction in FI compared to control AAV treated animals, indicative of an increase in energy expenditure. Fat and lean mass declined consistently in the *Glp1r* AAV treated groups compared to controls (Figure 1G). The BW change induced by semaglutide treatment correlated well to hepatic *Glp1r* gene expression (Figure 1H). These data support the conclusion that a GLP-1R ectopically expressed in the liver can functionally mimic the effects of GcgR agonism with respect to glucose excursion and FI-independent weight reduction in rodents.

**Supplemental Figure 2:**
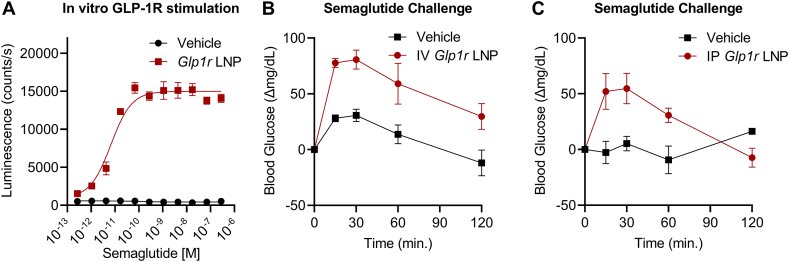
An LNP + mRNA construct successfully confers GLP-1R activity in hepatocytes of GLP-1R KO mice. (A) *In vitro* cAMP accumulation induced by semaglutide in cells transfected with GLP-1R via LNP. (B,C) *In vivo* stimulation of blood glucose in GLP-1R KO mice with GLP-1R reintroduction via LNP delivered by either (B) intravenous or (C) intraperitoneal injection. All data are presented as mean ± SEM.

We also utilized a lipid nanoparticle (LNP) encapsulating an mRNA construct to successfully drive *in vitro* (Supplemental Fig. 2A) and *in vivo* (Supplemental Fig. 2B and C) GLP-1R expression, as this modality may have more translational value. Semaglutide-mediated blood glucose excursion (Supplemental Fig. 2B and C) was increased in DIO GLP-1R KO mice treated with this *Glp1r* expressing LNP construct, although at a reduced magnitude compared to the AAV construct. While a demonstration of weight loss using the LNP construct would be valuable, the short duration of action for linear mRNAs makes this unfeasible in this body of work. Future technology development will focus on extending the duration of LNP delivered mRNA expression.

**Supplemental Figure 3:**
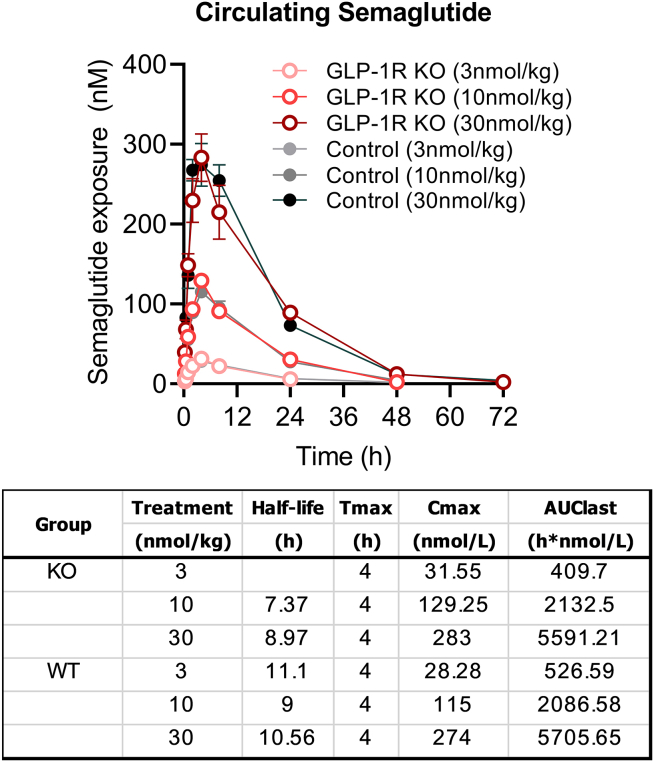
Endogenous GLP-1R does not modify the circulating PK profile of semaglutide. Circulating semaglutide profile in DIO WT and GLP-1R KO mice dosed acutely with semaglutide at 3 doses (3, 10, 30 nmol/kg); all data are presented as mean ± SEM.

Expanding from these findings, we tested whether the pharmacologic effects of dual GLP-1R/GcgR co-agonists to reduce FI and enhance EE can be replicated by co-treatment with the *Glp1r* AAV and semaglutide in DIO WT mice. The animals were dosed with either control or *Glp1r* AAV (10^12^ GC) on day −7 and began treatment with either vehicle or semaglutide (dose escalation from 1 to 100 nmol/kg) on day 0. Unexpectedly, the semaglutide and semaglutide + *Glp1r* AAV groups show similar BW, fat mass, and lean mass loss (Figure 2A,C,D) at doses of 1–30 nmol/kg semaglutide (Figure 2A; day 0–45). It is noteworthy that there is minimal FI reduction in the semaglutide + *Glp1r* AAV during this period compared to control AAV or *Glp1r* AAV alone (Figure 2B; day 0–45). However, at 100 nmol/kg semaglutide, we observe a stark decline in BW concomitant with reduced FI for the semaglutide + *Glp1r* AAV group compared to the group treated with semaglutide alone. Further, semaglutide + *Glp1r* AAV treated mice exhibit increased EE compared to semaglutide treatment alone in an acute setting (Figure 2E). These observations collectively suggest that lower doses of semaglutide (1–30 nmol/kg) drive BW reduction through two different mechanisms in the *Glp1r* AAV and control AAV treated mice. Mice treated with control AAV + semaglutide alone achieve BW loss via reduced FI as expected based on previous reports [8,26]. However, in mice treated with *Glp1r* AAV, semaglutide at lower doses (1–30 nmol/kg) acts primarily via the ectopic hepatic receptor pool to drive BW loss via energy expenditure (i.e. GLP-1 acting as glucagon). Further, we observe that semaglutide treatment alone acutely increases HR in mice with no effect on mean arterial pressure (MAP; Figure 2H,I), while semaglutide + *Glp1r* AAV intriguingly reduces this effect. We hypothesized that the modified pharmacology of semaglutide at lower doses (i.e. increased EE but no effect on FI, IPGTT, or HR at doses ≤30 nmol/kg) is due to ectopic GLP-1R mediated drug clearance. *In vitro* GLP-1R internalization by semaglutide is comparable to native GLP-1 (Figure 2J), suggesting a mechanism for ectopic GLP-1R to drive drug clearance. Endogenous GLP-1R populations do not facilitate a significant degree of semaglutide clearance (Supplemental Fig. 3); however, the additional pool of GLP-1R in the liver created by *Glp1r* AAV treatment facilitates a significant increase in semaglutide clearance compared to control groups (Figure 2K). Thus, the ectopic GLP-1R scavenges semaglutide from circulation and hinders its availability to reduce food intake (Figure 2B). This proposed model accounts for the lack of superior efficacy between groups until a sufficiently high dose of semaglutide is reached to overcome clearance by the ectopic receptor. From these data, we conclude that ectopic hepatic GLP-1R agonism via semaglutide in DIO mice can drive enhanced EE and additional BW loss compared to GLP-1R agonism alone without deleterious increases in HR. However, the full potential of this approach is obviated by ectopic GLP-1R mediated drug clearance requiring increased doses of the GLP-1R agonist.

Because there is an apparent clearance of semaglutide via internalization of the ectopic, hepatic GLP-1R, we hypothesized that a GLP-1R agonist which displays reduced ligand-mediated receptor internalization would circumvent this mechanism. We selected an acylated GLP-1 analogue with comparable protraction to semaglutide that induces a low degree of receptor internalization (NNC5840; Figure 3A) for testing in rodents [26]. NNC5840 showed a 1.9-fold reduction in circulating exposure in *Glp1r* AAV treated mice compared to controls, whereas semaglutide displayed a 6.6-fold reduction (Figure 3B). Repeated dosing of NNC5840 in DIO mice pre-treated with *Glp1r* AAV resulted in superior BW reduction and, critically, FI reduction (Figure 3C,D) compared to controls treated with NNC5840 alone, while semaglutide treatment with or without *Glp1r* AAV recapitulated our findings from previous studies (Figure 2). Accordingly, EE was also acutely elevated by NNC5840 in *Glp1r* AAV treated animals compared to control mice in a separate experiment (Figure 3E). Blood glucose during an IPGTT on day 0 was similar between control AAV and *Glp1r* AAV treated mice given NNC5840 (Figure 3F). These data support the hypothesis that a GLP-1R mono-agonist with low ligand-mediated receptor internalization (e.g. NNC5840) can limit the target mediated clearance mechanism presented by ectopic GLP-1R expression. Furthermore, combining GLP-1R agonists like NNC5840 with ectopic GLP-1R expression provides greater BW reducing efficacy than the GLP-1R agonist alone by engaging multiple mechanisms of action (MoA) including FI reduction and EE enhancement.

Finally, we tested the hypothesis that ectopic GLP-1R agonism can be leveraged to improve the efficacy of dual GLP-1R/GIPR agonists as a surrogate for triagonism. We began by demonstrating that our previously published dual incretin receptor agonist [8] exhibits low GLP-1R internalization *in vitro* (Figure 4A). These data are consistent with other dual GLP-1R/GIPR agonists including tirzepatide [27] and suggest our tool compound will be able to avoid the pharmacokinetic (PK) liabilities of coupling highly internalizing agonists like semaglutide with ectopic receptor expression. The dual GLP-1R/GIPR agonist + *Glp1r* AAV treatment was able to induce greater BW reduction in DIO WT mice than dual GLP-1R/GIPR agonist + control AAV (Figure 4B). These data suggest a mechanism by which activation of liver GPCRs act to reduce FI, but also do not necessarily rule out the hypothesized action of glucagon to reduce FI via a central mechanism. Additionally, we see a significant elevation in EE during acute dual GLP-1R/GIPR agonist treatment in the *Glp1r* AAV treated animals compared to controls (Figure 4D). We note a deterioration of glucose control in *Glp1r* AAV treated mice after 14 days of treatment compared to controls (Figure 4E). Finally, there is no additional increase in HR (Figure 4F) and no changes in MAP (Figure 4G) when combining *Glp1r* AAV with the dual receptor agonist at a dose (1 nmol/kg) that drives superior BW loss to the dual receptor agonist with control AAV. This indicates that the increased energy expenditure is not inherently linked to the CV liabilities of GcgR agonism, as has been hypothesized [28].

## 4 Discussion

These results lead to several conclusions that advance our goal of developing poly-pharmacologic approaches to treat obesity. The first is that functional GPCRs can be expressed ectopically to facilitate a poly-pharmacological platform. The *Glp1r* AAV and LNP/mRNA modalities employed in these studies drive expression of a functional GLP-1R in the liver of rodent models. The ectopic GLP-1R was activated by endogenous proglucagon products in lean mice (Supplemental Fig. 1), where it served to increase blood glucose in a variety of experimental paradigms, functionally recapitulating physiological GcgR agonism. In GLP-1R KO mice, the ectopic hepatic receptor increased glucose excursion in response to semaglutide (Figure 1). This effect was not seen in control GLP-1R KO mice and is the opposite response observed for semaglutide in a WT animal. Finally, we see that ectopic, hepatic GLP-1R agonism drives an increase in energy expenditure in response to GLP-1R and dual GLP-1R/GIPR agonists, an effect not observed with these compounds in WT animals [8]. This is interesting insofar as simply expressing a foreign Gαs-coupled GPCR in the hepatocyte and activating that receptor with a suitable ligand appears to be sufficient to replicate the beneficial pharmacology of GcgR for body weight reduction without the deleterious effects of chronic GcgR pharmacotherapy on HR. It appears the hepatocyte has the necessary machinery to express the GLP-1R, transport it to the cell membrane, and enable signal transduction, as we did no modification of the *mGlp1r* gene to facilitate these functions. This speaks to the notion that this approach is generalizable to other cell signalling systems much like DREADD technology [29]. Thus, cell-specific ectopic expression of receptors may offer a novel platform to drive a specific cell signalling cascade in addition to the cell- and tissue-targeting approaches currently being developed across the pharmaceutical industry.

Second, agonism of the ectopic, hepatic GLP-1R creates a novel mechanism of drug clearance and may have effects on biodistribution. Endogenous GLP-1R does not regulate semaglutide pharmacokinetics, and semaglutide clearance is increased with ectopic GLP-1R expression. This clearance mechanism can be overcome by introducing a GLP-1R ligand that displays low ligand-mediated receptor internalization. It is increasingly appreciated that GLP-1R agonists with a low receptor internalising profile [26,[30], [31], [32]], including tirzepatide [27], provide superior weight loss and glucose lowering efficacy. However, the ectopic GLP-1R expression system presented here offers another application for GLP-1R agonists with this particular *in vitro* profile, where it helps reduce drug clearance by the receptor. Additionally, these data indicate that agonists which induce low receptor internalization may prove particularly useful in systems where endogenous receptor mediated drug clearance is in play.

Third, agonism of the ectopic, hepatic GLP-1R with either semaglutide or a dual GLP-1R/GIPR agonist drive deleterious pharmacologic effects. Ectopic hepatic GLP-1R agonism combined with semaglutide and the dual GLP-1R/GIPR agonist did does not increase HR over that seen with the agonist alone. The tachycardic effect of semaglutide is decreased in *Glp1r* AAV treated animals compared to controls; however, this occurs at doses which do not induce superior weight loss and is likely the simple result of drug clearance by the ectopic GLP-1R. Yet, in the dual GLP-1R/GIPR agonist studies, we see no additional increase in HR in the *Glp1r* AAV treated mice above peptide alone at doses that induce EE and improve weight loss. It has been posited that increased EE observed with glucagon pharmacology is inherently linked to increased HR [28]; however, these data demonstrate that increases in EE and HR can be decoupled. In fact, the decreased tachycardic effect of semaglutide in *Glp1r* AAV treated mice suggests it is circulating exposure of the agonist that is more closely linked to these effects, rather than a secondary phenomenon driven by EE. One could hypothesize that the ectopic receptor approach, which increases the weight-lowering effect of semaglutide while mitigating the transient tachycardia, could amplify the known CV protective effects of the drug by decreasing stress on the heart itself and reducing peripheral arterial resistance via weight loss. Additionally, while ectopic receptor expression worsened glucose control acutely both with and without pharmacologic agonist (Figure 2F), chronic treatment led to an improvement in glucose control when comparing *Glp1r* AAV + agonist vs. *Glp-1r* AAV + vehicle (Figure 2G). This effect is likely due to the superior reductions in body weight between these two groups (Figure 2A). Conversely, it should be noted that the improvement in glucose control seen with control AAV + dual GLP-1R/GIPR agonist was greater than that seen with *Glp1r* AAV + dual GLP-1R/GIPR agonist (Figure 4E), indicating some blunting of the glycemic benefit of the drug alone. However, this is not seen for the control AAV + semaglutide compared to *Glp1r* AAV + semaglutide (Figure 2F), possibly due to the specific pharmacology or the duration of treatment.

In conclusion, these data support the development of a new methodology to safely introduce poly-pharmacology for the treatment of obesity. The ectopic, hepatic expression of GLP-1R and agonism of this receptor using semaglutide, NNC5840, or a dual GLP-1R/GIPR agonist imbues these molecules with novel EE enhancing efficacy that improves weight loss with no additional increase in HR. This approach of ectopically expressing and activating receptors with clinically validated pharmacophores represents a novel mechanism for engaging poly-pharmacology to treat obesity and perhaps other indications.

## CRediT authorship contribution statement

**Jonathan D. Douros:** Writing – original draft, Project administration, Methodology, Formal analysis, Data curation, Conceptualization. **Megan Capozzi:** Writing – original draft, Methodology, Investigation, Formal analysis, Conceptualization. **Aaron Novikoff:** Writing – original draft, Methodology, Investigation, Formal analysis, Data curation. **Jacek Mokrosinski:** Writing – original draft, Methodology, Investigation, Formal analysis, Data curation, Conceptualization. **Barent DuBois:** Writing – original draft, Methodology, Investigation, Formal analysis, Data curation, Conceptualization. **Joseph Stock:** Writing – original draft, Methodology, Investigation. **Rebecca Rohlfs:** Writing – original draft, Project administration, Methodology, Investigation, Data curation. **Mikayla Anderson:** Writing – original draft, Methodology, Investigation, Formal analysis. **Dominika J. Jedrzejcyk:** Writing – original draft, Methodology, Investigation, Formal analysis, Conceptualization. **Svend Poulsen:** Writing – original draft, Methodology, Investigation, Formal analysis, Data curation. **Erik Oude Blenke:** Writing – original draft, Methodology, Investigation, Formal analysis, Conceptualization. **Tomas Dago:** Writing – original draft, Methodology, Investigation, Formal analysis, Data curation. **Kasper Huus:** Writing – original draft, Project administration, Methodology, Investigation, Formal analysis. **Peder L. Nørby:** Writing – original draft, Methodology, Investigation, Formal analysis. **Sune Kobberup:** Writing – original draft, Project administration, Methodology, Investigation, Data curation, Conceptualization. **Marita Rivir:** Writing – original draft, Visualization, Methodology, Investigation, Formal analysis. **Joyce Sorrell:** Writing – original draft, Visualization, Project administration, Methodology, Investigation, Formal analysis. **Stephanie A. Mowery:** Writing – original draft, Visualization, Methodology, Investigation, Formal analysis. **Daniel J. Drucker:** Writing – original draft, Resources, Project administration. **David A. D'Alessio:** Writing – original draft, Supervision, Resources, Project administration, Conceptualization. **Jonathan E. Campbell:** Writing – original draft, Resources, Project administration, Data curation, Conceptualization. **Timo D. Müller:** Writing – original draft, Supervision, Resources, Project administration, Formal analysis, Data curation. **Diego Perez-Tilve:** Writing – original draft, Supervision, Resources, Project administration, Formal analysis, Data curation. **Brian Finan:** Writing – original draft, Supervision, Resources, Project administration, Conceptualization. **Patrick J. Knerr:** Writing – original draft, Visualization, Validation, Supervision, Project administration, Methodology, Investigation, Formal analysis.

## Declaration of competing interest

JDD, JM, BD, JS, RR, JA, DJJ, SP, EOB, KH, PLN, SK, SAM, BF, and PJK are or were employees of Novo Nordisk while this work was performed. JDD, SAM, and PJK receive research funding from Eli Lilly and are cofounders and shareholders in Volari Therapeutics, both unrelated to this work. BF is currently an employee of Eli Lilly. D.P.-T. maintains research collaborations with Novo Nordisk, MBX Biosciences and BlueWater Biosciences, and is a minority shareholder of BlueWater Biosciences. JEC and DAD receive research support from Eli Lilly and Novo Nordisk to carry out basic science in this area and serve as advisors for Structure Therapeutics. DAD serves as an advisor for Eli Lilly. TDM receives research funding from Novo Nordisk and speaking fees from Eli Lilly, Novo Nordisk, Mercodia, AstraZeneca, Berlin Chemie and Sanofi-Aventis. Research funding for this work was provided by Novo Nordisk.

## Data Availability

No data was used for the research described in the article.
